# Necrology

**Published:** 1897-03

**Authors:** 


### NECROLOGY.

At New Bedford, Mass., on Sunday morning, January 3d, after an illness of two months, O. H. Flagg, M.D., V.S., quietly passed away. Born at Barre, Mass., February 28, 1823, he had nearly attained the age of seventy-four years. At the age of thirty he had entered upon the study of human medicine, taking his degree at a Cincinnati medical school; but, following the more natural bent of his inclinations, he entered the college of Dr. George H. Dadd, from which school in 1859 he was graduated and at once began practice as a veterinarian, which won for him the highest honors and esteem among the people with whom he lived and for whom he labored. He was a charter member of the United States Veterinary Medical Association, with which he retained his allegiance until his death. He was a member of the Massachusetts Veterinary Medical Association and was endeared to every member of the profession who was favored with his acquaintance.

J. D. Rutherford, M.D., V.S., Principal of the Detroit Veterinary College, died very suddenly on the evening of January 11th, of rheumatism of the heart. Dr. Rutherford was a graduate of Ontario Veterinary College, class of 1887; also of the Rush Medical College of Chicago. At the time of his death, though engrossed in the work of the college, he was pursuing the study of law. The doctor succeeded Dr. S. Brenton as the principal of the Veterinary Department at Detroit, and his death makes the second loss of its chief director in the past two years.

David L. Lancaster, a well-known veterinary surgeon of Shelbyville, Indiana, died last month, aged sixty years.

C. A. Turley, V.S., Brandon, Manitoba, died recently. He was a graduate of the Ontario Veterinary College, class of 1895.

Dr. Michael Gallagher, a registered non-graduate of Pittsburg, Pa., died in February at the age of forty-four years.

At Allentown, Pa., on February 15, 1897, Ellen S. Foelker, and on February 19, 1897, Rosa A. E. Foelker, daughters of John C. Foelker, V.S.

